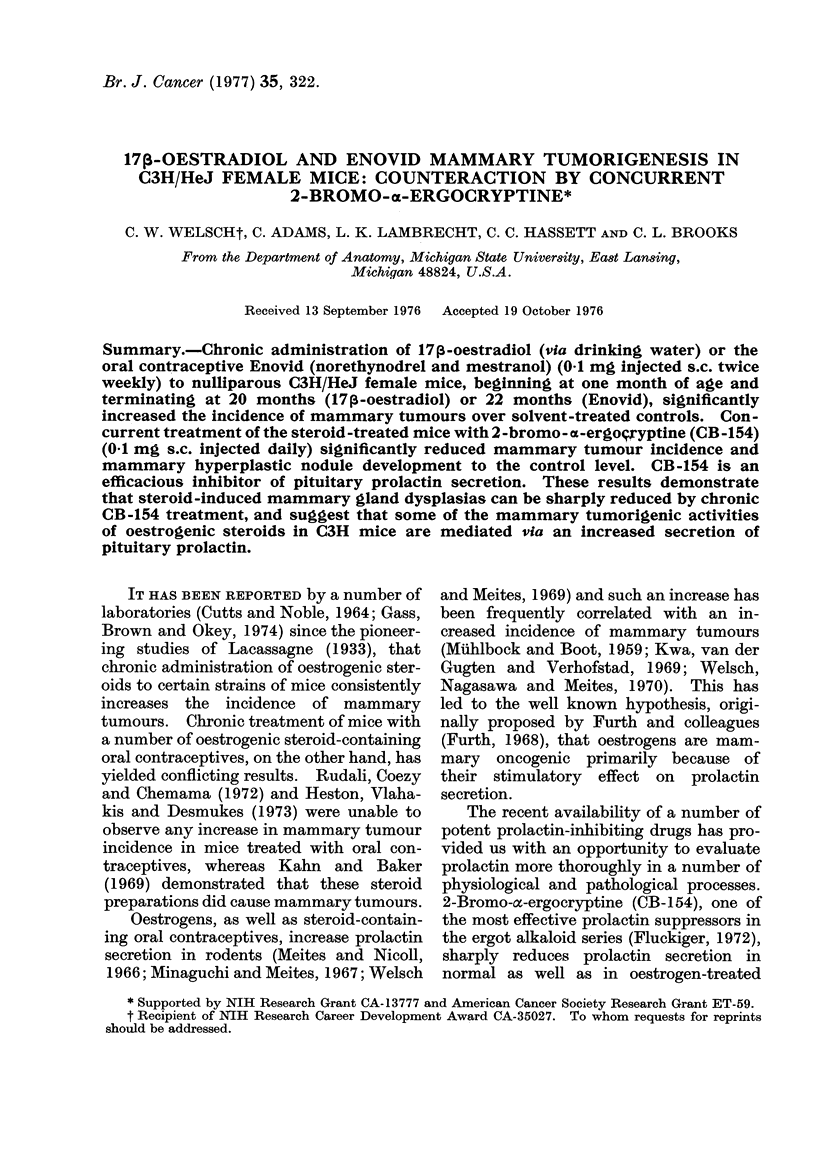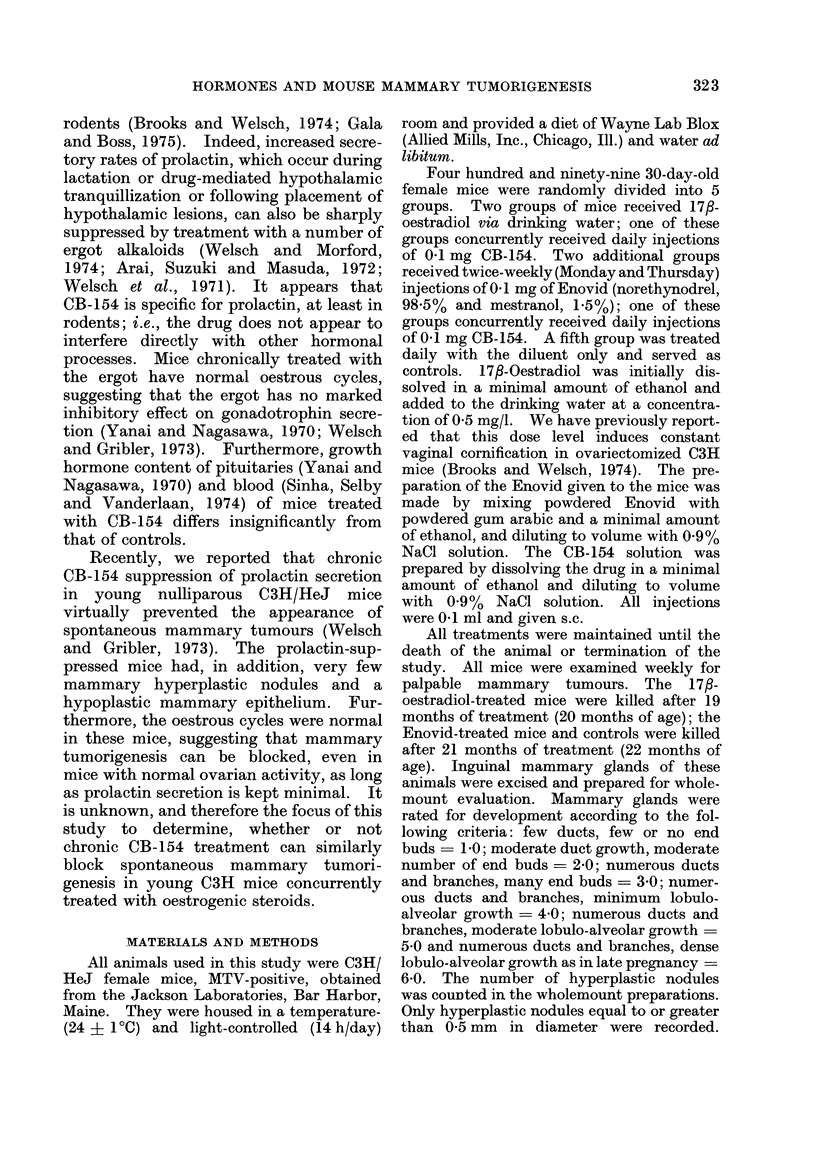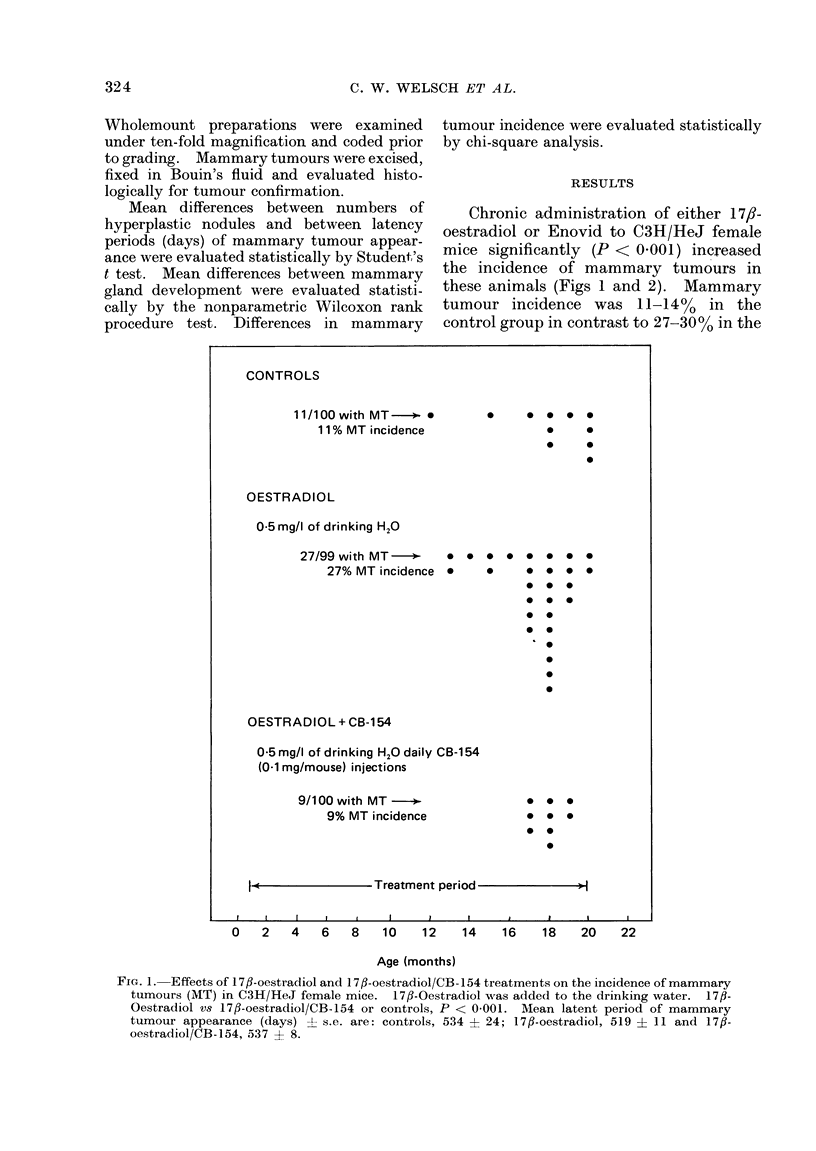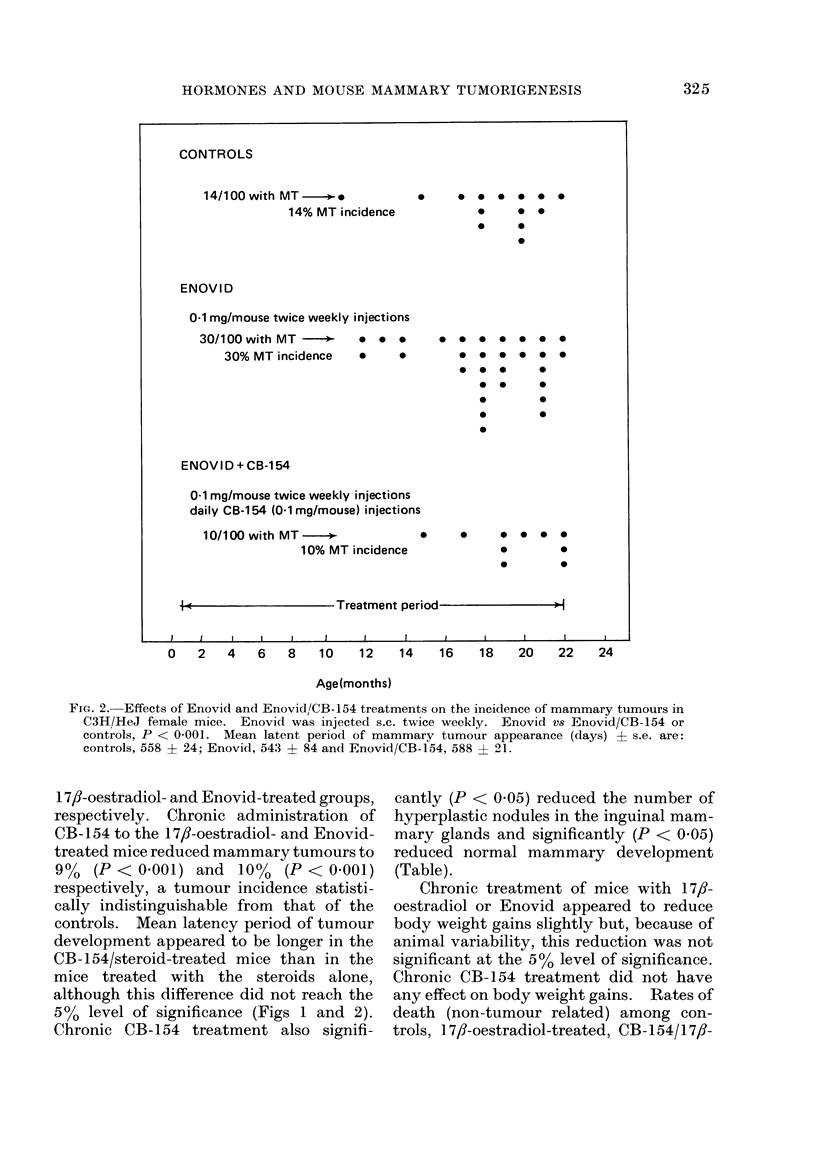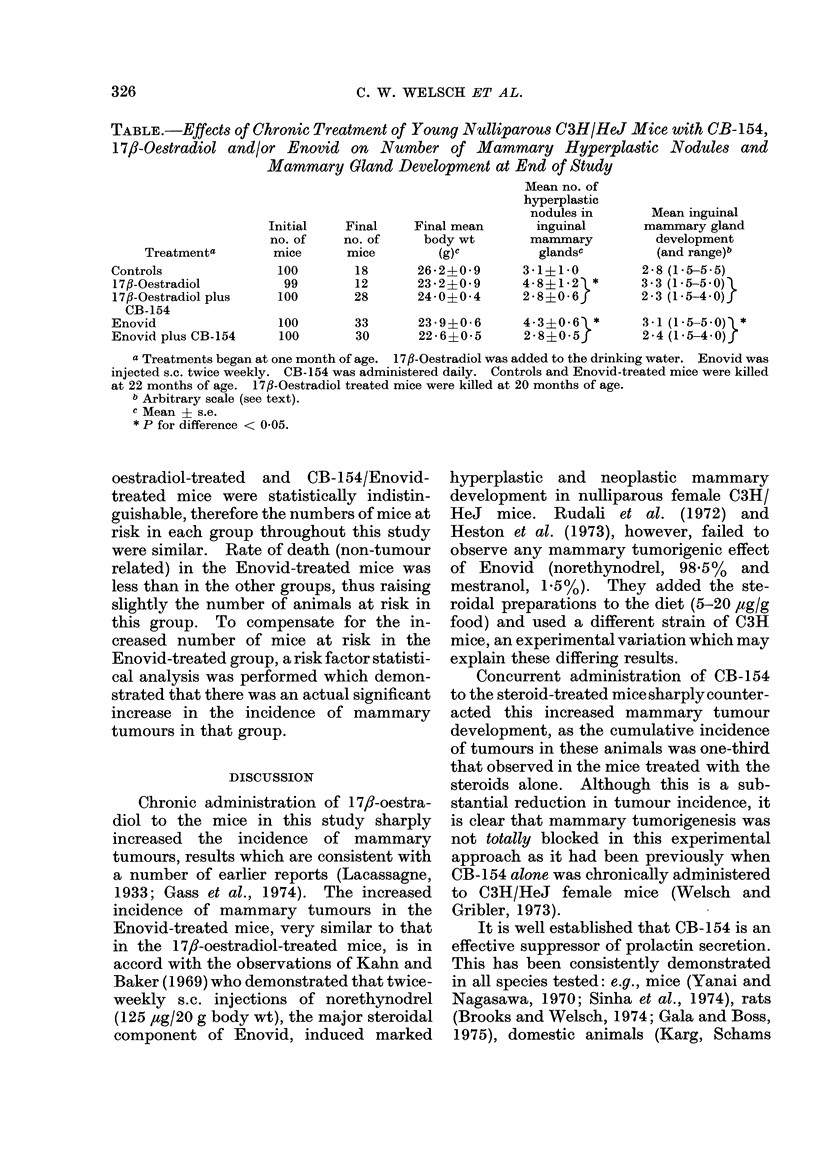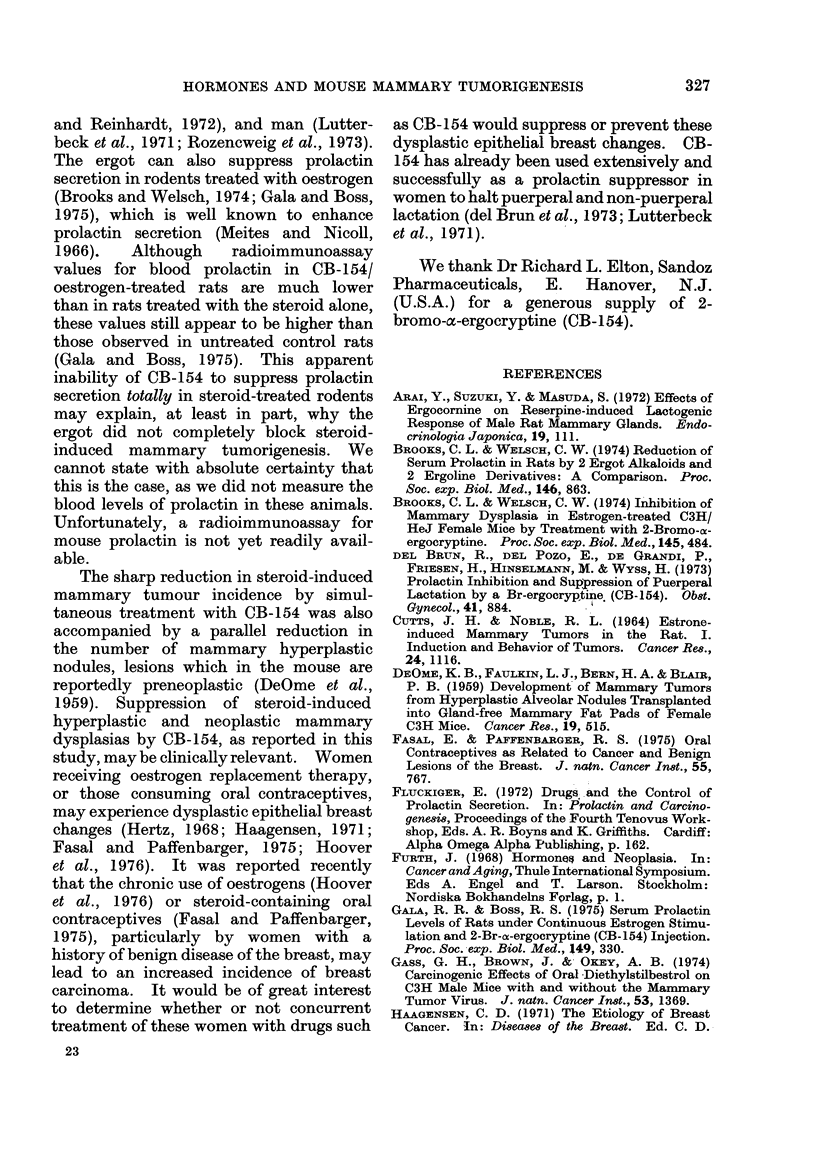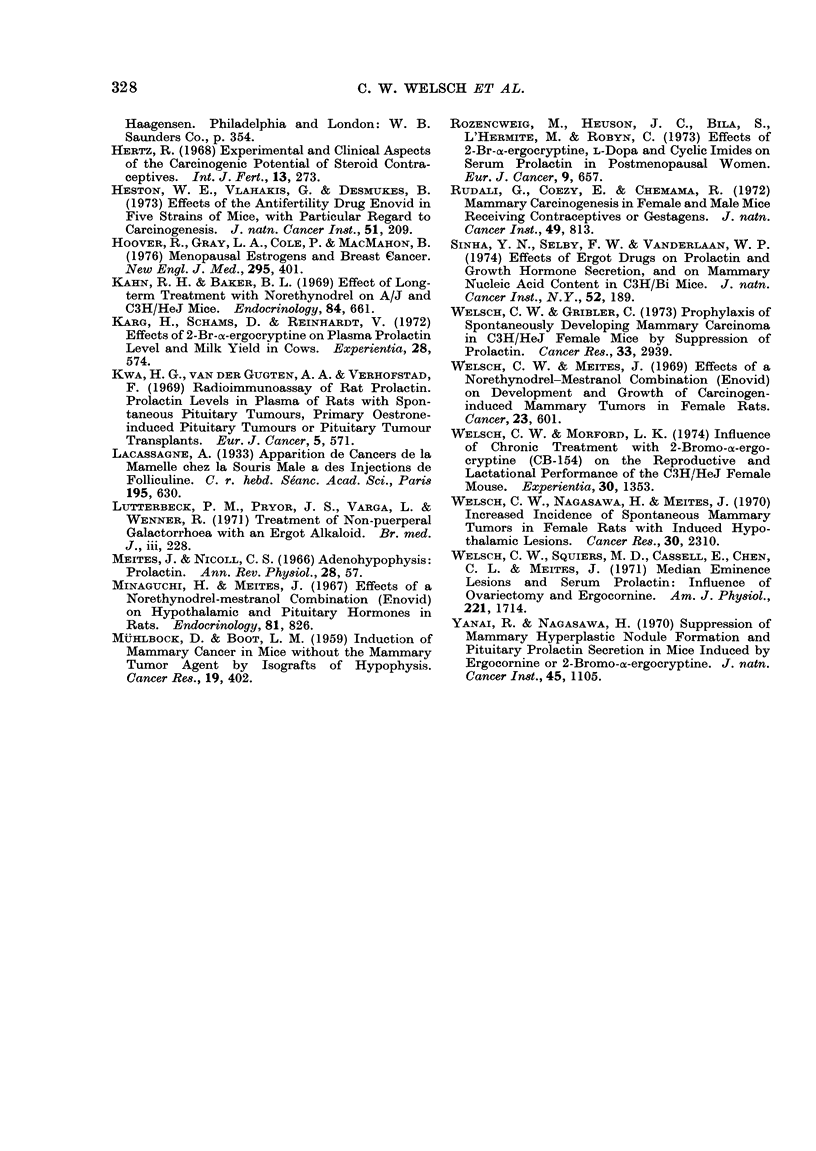# 17beta-oestradiol and Enovid mammary tumorigenesis in C3H/HeJ female mice: counteraction by concurrent 2-bromo-alpha-ergocryptine.

**DOI:** 10.1038/bjc.1977.46

**Published:** 1977-03

**Authors:** C. W. Welsch, C. Adams, L. K. Lambrecht, C. C. Hassett, C. L. Brooks

## Abstract

Chronic administration of 17beta-oestradiol (via drinking water) or the oral contraceptive Enovid (norethynodrel and mestranol) (0-1 mg injected s.c. twice weekly) to nulliparous C3H/HeJ female mice, beginning at one month of age and terminating at 20 months (17beta-oestradiol) or 22 months (Enovid), significantly increased the incidence of mammary tumours over solvent-treated controls. Concurrent treatment of the steroid-treated mice with 2-bromo-alpha-ergocryptine (CB-154) (0-1 mg s.c. injected daily) significantly reduced mammary tumour incidence and mammary hyperplastic nodule development to the control level. CB-154 is an efficacious inhibitor of pituitary prolactin secretion. These results demonstrate that steroid-induced mammary gland dysplasias can be sharply reduced by chronic CB-154 treatment, and suggest that some of the mammary tumorigenic activities of oestrogenic steroids in C3H mice are mediated via an increased secretion of pituitary prolactin.


Br. J. Cancer (1977) 35, 322.

### 17β-OESTRADIOL AND ENOVID MAMMARY TUMORIGENESIS IN C3H/HeJ FEMALE MICE: COUNTERACTION BY CONCURRENT 2-BROMO-α-ERGOCRYPTINE*

C. W. WELSCH†, C. ADAMS, L. K. LAMBRECHT, C. C. HASSETT AND C. L. BROOKS

*From the Department of Anatomy, Michigan State University, East Lansing, Michigan 48824, U.S.A.*

Received 13 September 1976 Accepted 19 October 1976

**Summary.—Chronic administration of 17β-oestradiol (*via* drinking water) or the oral contraceptive Enovid (norethynodrel and mestranol) (0·1 mg injected s.c. twice weekly) to nulliparous C3H/HeJ female mice, beginning at one month of age and terminating at 20 months (17β-oestradiol) or 22 months (Enovid), significantly increased the incidence of mammary tumours over solvent-treated controls. Concurrent treatment of the steroid-treated mice with 2-bromo-α-ergocryptine (CB-154) (0·1 mg s.c. injected daily) significantly reduced mammary tumour incidence and mammary hyperplastic nodule development to the control level. CB-154 is an efficacious inhibitor of pituitary prolactin secretion. These results demonstrate that steroid-induced mammary gland dysplasias can be sharply reduced by chronic CB-154 treatment, and suggest that some of the mammary tumorigenic activities of oestrogenic steroids in C3H mice are mediated *via* an increased secretion of pituitary prolactin.**

IT HAS BEEN REPORTED by a number of laboratories (Cutts and Noble, 1964; Gass, Brown and Okey, 1974) since the pioneering studies of Lacassagne (1933), that chronic administration of oestrogenic steroids to certain strains of mice consistently increases the incidence of mammary tumours. Chronic treatment of mice with a number of oestrogenic steroid-containing oral contraceptives, on the other hand, has yielded conflicting results. Rudali, Coezy and Chemama (1972) and Heston, Vlahakis and Desmukes (1973) were unable to observe any increase in mammary tumour incidence in mice treated with oral contraceptives, whereas Kahn and Baker (1969) demonstrated that these steroid preparations did cause mammary tumours.

Oestrogens, as well as steroid-containing oral contraceptives, increase prolactin secretion in rodents (Meites and Nicoll, 1966; Minaguchi and Meites, 1967; Welsch and Meites, 1969) and such an increase has been frequently correlated with an increased incidence of mammary tumours (Mühlbock and Boot, 1959; Kwa, van der Gugten and Verhofstad, 1969; Welsch, Nagasawa and Meites, 1970). This has led to the well known hypothesis, originally proposed by Furth and colleagues (Furth, 1968), that oestrogens are mammary oncogenic primarily because of their stimulatory effect on prolactin secretion.

The recent availability of a number of potent prolactin-inhibiting drugs has provided us with an opportunity to evaluate prolactin more thoroughly in a number of physiological and pathological processes. 2-Bromo-α-ergocryptine (CB-154), one of the most effective prolactin suppressors in the ergot alkaloid series (Fluckiger, 1972), sharply reduces prolactin secretion in normal as well as in oestrogen-treated

* Supported by NIH Research Grant CA-13777 and American Cancer Society Research Grant ET-59.
† Recipient of NIH Research Career Development Award CA-35027. To whom requests for reprints should be addressed.

rodents (Brooks and Welsch, 1974; Gala and Boss, 1975). Indeed, increased secretory rates of prolactin, which occur during lactation or drug-mediated hypothalamic tranquillization or following placement of hypothalamic lesions, can also be sharply suppressed by treatment with a number of ergot alkaloids (Welsch and Morford, 1974; Arai, Suzuki and Masuda, 1972; Welsch *et al.*, 1971). It appears that CB-154 is specific for prolactin, at least in rodents; *i.e.*, the drug does not appear to interfere directly with other hormonal processes. Mice chronically treated with the ergot have normal oestrous cycles, suggesting that the ergot has no marked inhibitory effect on gonadotrophin secretion (Yanai and Nagasawa, 1970; Welsch and Gribler, 1973). Furthermore, growth hormone content of pituitaries (Yanai and Nagasawa, 1970) and blood (Sinha, Selby and Vanderlaan, 1974) of mice treated with CB-154 differs insignificantly from that of controls.

Recently, we reported that chronic CB-154 suppression of prolactin secretion in young nulliparous C3H/HeJ mice virtually prevented the appearance of spontaneous mammary tumours (Welsch and Gribler, 1973). The prolactin-suppressed mice had, in addition, very few mammary hyperplastic nodules and a hypoplastic mammary epithelium. Furthermore, the oestrous cycles were normal in these mice, suggesting that mammary tumorigenesis can be blocked, even in mice with normal ovarian activity, as long as prolactin secretion is kept minimal. It is unknown, and therefore the focus of this study to determine, whether or not chronic CB-154 treatment can similarly block spontaneous mammary tumorigenesis in young C3H mice concurrently treated with oestrogenic steroids.

### MATERIALS AND METHODS

All animals used in this study were C3H/HeJ female mice, MTV-positive, obtained from the Jackson Laboratories, Bar Harbor, Maine. They were housed in a temperature- (24 ± 1 °C) and light-controlled (14 h/day) room and provided a diet of Wayne Lab Blox (Allied Mills, Inc., Chicago, Ill.) and water *ad libitum*.

Four hundred and ninety-nine 30-day-old female mice were randomly divided into 5 groups. Two groups of mice received 17β-oestradiol *via* drinking water; one of these groups concurrently received daily injections of 0·1 mg CB-154. Two additional groups received twice-weekly (Monday and Thursday) injections of 0·1 mg of Enovid (norethynodrel, 98·5% and mestranol, 1·5%); one of these groups concurrently received daily injections of 0·1 mg CB-154. A fifth group was treated daily with the diluent only and served as controls. 17β-Oestradiol was initially dissolved in a minimal amount of ethanol and added to the drinking water at a concentration of 0·5 mg/l. We have previously reported that this dose level induces constant vaginal cornification in ovariectomized C3H mice (Brooks and Welsch, 1974). The preparation of the Enovid given to the mice was made by mixing powdered Enovid with powdered gum arabic and a minimal amount of ethanol, and diluting to volume with 0·9% NaCl solution. The CB-154 solution was prepared by dissolving the drug in a minimal amount of ethanol and diluting to volume with 0·9% NaCl solution. All injections were 0·1 ml and given s.c.

All treatments were maintained until the death of the animal or termination of the study. All mice were examined weekly for palpable mammary tumours. The 17β-oestradiol-treated mice were killed after 19 months of treatment (20 months of age); the Enovid-treated mice and controls were killed after 21 months of treatment (22 months of age). Inguinal mammary glands of these animals were excised and prepared for wholemount evaluation. Mammary glands were rated for development according to the following criteria: few ducts, few or no end buds = 1·0; moderate duct growth, moderate number of end buds = 2·0; numerous ducts and branches, many end buds = 3·0; numerous ducts and branches, minimum lobulo-alveolar growth = 4·0; numerous ducts and branches, moderate lobulo-alveolar growth = 5·0 and numerous ducts and branches, dense lobulo-alveolar growth as in late pregnancy = 6·0. The number of hyperplastic nodules was counted in the wholemount preparations. Only hyperplastic nodules equal to or greater than 0·5 mm in diameter were recorded.

Wholemount preparations were examined under ten-fold magnification and coded prior to grading. Mammary tumours were excised, fixed in Bouin's fluid and evaluated histologically for tumour confirmation.

Mean differences between numbers of hyperplastic nodules and between latency periods (days) of mammary tumour appearance were evaluated statistically by Student's *t* test. Mean differences between mammary gland development were evaluated statistically by the nonparametric Wilcoxon rank procedure test. Differences in mammary tumour incidence were evaluated statistically by chi-square analysis.

### RESULTS

Chronic administration of either 17β-oestradiol or Enovid to C3H/HeJ female mice significantly ($P < 0{\cdot}001$) increased the incidence of mammary tumours in these animals (Figs 1 and 2). Mammary tumour incidence was 11–14% in the control group in contrast to 27–30% in the

FIG. 1.—Effects of 17β-oestradiol and 17β-oestradiol/CB-154 treatments on the incidence of mammary tumours (MT) in C3H/HeJ female mice. 17β-Oestradiol was added to the drinking water. 17β-Oestradiol *vs* 17β-oestradiol/CB-154 or controls, $P < 0{\cdot}001$. Mean latent period of mammary tumour appearance (days) ± s.e. are: controls, 534 ± 24; 17β-oestradiol, 519 ± 11 and 17β-oestradiol/CB-154, 537 ± 8.

FIG. 2.—Effects of Enovid and Enovid/CB-154 treatments on the incidence of mammary tumours in C3H/HeJ female mice. Enovid was injected s.c. twice weekly. Enovid *vs* Enovid/CB-154 or controls, $P < 0{\cdot}001$. Mean latent period of mammary tumour appearance (days) $\pm$ s.e. are: controls, 558 $\pm$ 24; Enovid, 543 $\pm$ 84 and Enovid/CB-154, 588 $\pm$ 21.

17β-oestradiol- and Enovid-treated groups, respectively. Chronic administration of CB-154 to the 17β-oestradiol- and Enovid-treated mice reduced mammary tumours to 9% ($P < 0{\cdot}001$) and 10% ($P < 0{\cdot}001$) respectively, a tumour incidence statistically indistinguishable from that of the controls. Mean latency period of tumour development appeared to be longer in the CB-154/steroid-treated mice than in the mice treated with the steroids alone, although this difference did not reach the 5% level of significance (Figs 1 and 2). Chronic CB-154 treatment also significantly ($P < 0{\cdot}05$) reduced the number of hyperplastic nodules in the inguinal mammary glands and significantly ($P < 0{\cdot}05$) reduced normal mammary development (Table).

Chronic treatment of mice with 17β-oestradiol or Enovid appeared to reduce body weight gains slightly but, because of animal variability, this reduction was not significant at the 5% level of significance. Chronic CB-154 treatment did not have any effect on body weight gains. Rates of death (non-tumour related) among controls, 17β-oestradiol-treated, CB-154/17β-

TABLE.—*Effects of Chronic Treatment of Young Nulliparous C3H/HeJ Mice with CB*-154, 17β-*Oestradiol and/or Enovid on Number of Mammary Hyperplastic Nodules and Mammary Gland Development at End of Study*

| Treatment[a] | Initial no. of mice | Final no. of mice | Final mean body wt (g)[c] | Mean no. of hyperplastic nodules in inguinal mammary glands[c] | Mean inguinal mammary gland development (and range)[b] |
|---|---|---|---|---|---|
| Controls | 100 | 18 | 26·2±0·9 | 3·1±1·0 | 2·8 (1·5–5·5) |
| 17β-Oestradiol | 99 | 12 | 23·2±0·9 | 4·8±1·2 } * | 3·3 (1·5–5·0) } |
| 17β-Oestradiol plus CB-154 | 100 | 28 | 24·0±0·4 | 2·8±0·6 } | 2·3 (1·5–4·0) } |
| Enovid | 100 | 33 | 23·9±0·6 | 4·3±0·6 } * | 3·1 (1·5–5·0) } * |
| Enovid plus CB-154 | 100 | 30 | 22·6±0·5 | 2·8±0·5 } | 2·4 (1·5–4·0) } |

[a] Treatments began at one month of age. 17β-Oestradiol was added to the drinking water. Enovid was injected s.c. twice weekly. CB-154 was administered daily. Controls and Enovid-treated mice were killed at 22 months of age. 17β-Oestradiol treated mice were killed at 20 months of age.
[b] Arbitrary scale (see text).
[c] Mean ± s.e.
* $P$ for difference $< 0·05$.

oestradiol-treated and CB-154/Enovid-treated mice were statistically indistinguishable, therefore the numbers of mice at risk in each group throughout this study were similar. Rate of death (non-tumour related) in the Enovid-treated mice was less than in the other groups, thus raising slightly the number of animals at risk in this group. To compensate for the increased number of mice at risk in the Enovid-treated group, a risk factor statistical analysis was performed which demonstrated that there was an actual significant increase in the incidence of mammary tumours in that group.

### DISCUSSION

Chronic administration of 17β-oestradiol to the mice in this study sharply increased the incidence of mammary tumours, results which are consistent with a number of earlier reports (Lacassagne, 1933; Gass *et al.*, 1974). The increased incidence of mammary tumours in the Enovid-treated mice, very similar to that in the 17β-oestradiol-treated mice, is in accord with the observations of Kahn and Baker (1969) who demonstrated that twice-weekly s.c. injections of norethynodrel (125 μg/20 g body wt), the major steroidal component of Enovid, induced marked hyperplastic and neoplastic mammary development in nulliparous female C3H/HeJ mice. Rudali *et al.* (1972) and Heston *et al.* (1973), however, failed to observe any mammary tumorigenic effect of Enovid (norethynodrel, 98·5% and mestranol, 1·5%). They added the steroidal preparations to the diet (5–20 μg/g food) and used a different strain of C3H mice, an experimental variation which may explain these differing results.

Concurrent administration of CB-154 to the steroid-treated mice sharply counteracted this increased mammary tumour development, as the cumulative incidence of tumours in these animals was one-third that observed in the mice treated with the steroids alone. Although this is a substantial reduction in tumour incidence, it is clear that mammary tumorigenesis was not *totally* blocked in this experimental approach as it had been previously when CB-154 *alone* was chronically administered to C3H/HeJ female mice (Welsch and Gribler, 1973).

It is well established that CB-154 is an effective suppressor of prolactin secretion. This has been consistently demonstrated in all species tested: *e.g.*, mice (Yanai and Nagasawa, 1970; Sinha *et al.*, 1974), rats (Brooks and Welsch, 1974; Gala and Boss, 1975), domestic animals (Karg, Schams and Reinhardt, 1972), and man (Lutterbeck *et al.*, 1971; Rozencweig *et al.*, 1973). The ergot can also suppress prolactin secretion in rodents treated with oestrogen (Brooks and Welsch, 1974; Gala and Boss, 1975), which is well known to enhance prolactin secretion (Meites and Nicoll, 1966). Although radioimmunoassay values for blood prolactin in CB-154/oestrogen-treated rats are much lower than in rats treated with the steroid alone, these values still appear to be higher than those observed in untreated control rats (Gala and Boss, 1975). This apparent inability of CB-154 to suppress prolactin secretion *totally* in steroid-treated rodents may explain, at least in part, why the ergot did not completely block steroid-induced mammary tumorigenesis. We cannot state with absolute certainty that this is the case, as we did not measure the blood levels of prolactin in these animals. Unfortunately, a radioimmunoassay for mouse prolactin is not yet readily available.

The sharp reduction in steroid-induced mammary tumour incidence by simultaneous treatment with CB-154 was also accompanied by a parallel reduction in the number of mammary hyperplastic nodules, lesions which in the mouse are reportedly preneoplastic (DeOme *et al.*, 1959). Suppression of steroid-induced hyperplastic and neoplastic mammary dysplasias by CB-154, as reported in this study, may be clinically relevant. Women receiving oestrogen replacement therapy, or those consuming oral contraceptives, may experience dysplastic epithelial breast changes (Hertz, 1968; Haagensen, 1971; Fasal and Paffenbarger, 1975; Hoover *et al.*, 1976). It was reported recently that the chronic use of oestrogens (Hoover *et al.*, 1976) or steroid-containing oral contraceptives (Fasal and Paffenbarger, 1975), particularly by women with a history of benign disease of the breast, may lead to an increased incidence of breast carcinoma. It would be of great interest to determine whether or not concurrent treatment of these women with drugs such as CB-154 would suppress or prevent these dysplastic epithelial breast changes. CB-154 has already been used extensively and successfully as a prolactin suppressor in women to halt puerperal and non-puerperal lactation (del Brun *et al.*, 1973; Lutterbeck *et al.*, 1971).

We thank Dr Richard L. Elton, Sandoz Pharmaceuticals, E. Hanover, N.J. (U.S.A.) for a generous supply of 2-bromo-α-ergocryptine (CB-154).

### REFERENCES

Arai, Y., Suzuki, Y. & Masuda, S. (1972) Effects of Ergocornine on Reserpine-induced Lactogenic Response of Male Rat Mammary Glands. *Endocrinologia Japonica*, **19**, 111.

Brooks, C. L. & Welsch, C. W. (1974) Reduction of Serum Prolactin in Rats by 2 Ergot Alkaloids and 2 Ergoline Derivatives: A Comparison. *Proc. Soc. exp. Biol. Med.*, **146**, 863.

Brooks, C. L. & Welsch, C. W. (1974) Inhibition of Mammary Dysplasia in Estrogen-treated C3H/HeJ Female Mice by Treatment with 2-Bromo-α-ergocryptine. *Proc. Soc. exp. Biol. Med.*, **145**, 484.

del Brun, R., del Pozo, E., de Grandi, P., Friesen, H., Hinselmann, M. & Wyss, H. (1973) Prolactin Inhibition and Suppression of Puerperal Lactation by a Br-ergocryptine (CB-154). *Obst. Gynecol.*, **41**, 884.

Cutts, J. H. & Noble, R. L. (1964) Estrone-induced Mammary Tumors in the Rat. I. Induction and Behavior of Tumors. *Cancer Res.*, **24**, 1116.

DeOme, K. B., Faulkin, L. J., Bern, H. A. & Blair, P. B. (1959) Development of Mammary Tumors from Hyperplastic Alveolar Nodules Transplanted into Gland-free Mammary Fat Pads of Female C3H Mice. *Cancer Res.*, **19**, 515.

Fasal, E. & Paffenbarger, R. S. (1975) Oral Contraceptives as Related to Cancer and Benign Lesions of the Breast. *J. natn. Cancer Inst.*, **55**, 767.

Fluckiger, E. (1972) Drugs and the Control of Prolactin Secretion. In: *Prolactin and Carcinogenesis*, Proceedings of the Fourth Tenovus Workshop, Eds. A. R. Boyns and K. Griffiths. Cardiff: Alpha Omega Alpha Publishing, p. 162.

Furth, J. (1968) Hormones and Neoplasia. In: *Cancer and Aging*, Thule International Symposium. Eds A. Engel and T. Larson. Stockholm: Nordiska Bokhandelns Førlag, p. 1.

Gala, R. R. & Boss, R. S. (1975) Serum Prolactin Levels of Rats under Continuous Estrogen Stimulation and 2-Br-α-ergocryptine (CB-154) Injection. *Proc. Soc. exp. Biol. Med.*, **149**, 330.

Gass, G. H., Brown, J. & Okey, A. B. (1974) Carcinogenic Effects of Oral Diethylstilbestrol on C3H Male Mice with and without the Mammary Tumor Virus. *J. natn. Cancer Inst.*, **53**, 1369.

Haagensen, C. D. (1971) The Etiology of Breast Cancer. In: *Diseases of the Breast*. Ed. C. D.

Haagensen. Philadelphia and London: W. B. Saunders Co., p. 354.

HERTZ, R. (1968) Experimental and Clinical Aspects of the Carcinogenic Potential of Steroid Contraceptives. *Int. J. Fert.*, **13**, 273.

HESTON, W. E., VLAHAKIS, G. & DESMUKES, B. (1973) Effects of the Antifertility Drug Enovid in Five Strains of Mice, with Particular Regard to Carcinogenesis. *J. natn. Cancer Inst.*, **51**, 209.

HOOVER, R., GRAY, L. A., COLE, P. & MACMAHON, B. (1976) Menopausal Estrogens and Breast Cancer. *New Engl. J. Med.*, **295**, 401.

KAHN, R. H. & BAKER, B. L. (1969) Effect of Long-term Treatment with Norethynodrel on A/J and C3H/HeJ Mice. *Endocrinology*, **84**, 661.

KARG, H., SCHAMS, D. & REINHARDT, V. (1972) Effects of 2-Br-α-ergocryptine on Plasma Prolactin Level and Milk Yield in Cows. *Experientia*, **28**, 574.

KWA, H. G., VAN DER GUGTEN, A. A. & VERHOFSTAD, F. (1969) Radioimmunoassay of Rat Prolactin. Prolactin Levels in Plasma of Rats with Spontaneous Pituitary Tumours, Primary Oestrone-induced Pituitary Tumours or Pituitary Tumour Transplants. *Eur. J. Cancer*, **5**, 571.

LACASSAGNE, A. (1933) Apparition de Cancers de la Mamelle chez la Souris Male a des Injections de Folliculine. *C. r. hebd. Séanc. Acad. Sci., Paris* **195**, 630.

LUTTERBECK, P. M., PRYOR, J. S., VARGA, L. & WENNER, R. (1971) Treatment of Non-puerperal Galactorrhoea with an Ergot Alkaloid. *Br. med. J.*, iii, 228.

MEITES, J. & NICOLL, C. S. (1966) Adenohypophysis: Prolactin. *Ann. Rev. Physiol.*, **28**, 57.

MINAGUCHI, H. & MEITES, J. (1967) Effects of a Norethynodrel-mestranol Combination (Enovid) on Hypothalamic and Pituitary Hormones in Rats. *Endocrinology*, **81**, 826.

MÜHLBOCK, D. & BOOT, L. M. (1959) Induction of Mammary Cancer in Mice without the Mammary Tumor Agent by Isografts of Hypophysis. *Cancer Res.*, **19**, 402.

ROZENCWEIG, M., HEUSON, J. C., BILA, S., L'HERMITE, M. & ROBYN, C. (1973) Effects of 2-Br-α-ergocryptine, L-Dopa and Cyclic Imides on Serum Prolactin in Postmenopausal Women. *Eur. J. Cancer*, **9**, 657.

RUDALI, G., COEZY, E. & CHEMAMA, R. (1972) Mammary Carcinogenesis in Female and Male Mice Receiving Contraceptives or Gestagens. *J. natn. Cancer Inst.*, **49**, 813.

SINHA, Y. N., SELBY, F. W. & VANDERLAAN, W. P. (1974) Effects of Ergot Drugs on Prolactin and Growth Hormone Secretion, and on Mammary Nucleic Acid Content in C3H/Bi Mice. *J. natn. Cancer Inst., N.Y.*, **52**, 189.

WELSCH, C. W. & GRIBLER, C. (1973) Prophylaxis of Spontaneously Developing Mammary Carcinoma in C3H/HeJ Female Mice by Suppression of Prolactin. *Cancer Res.*, **33**, 2939.

WELSCH, C. W. & MEITES, J. (1969) Effects of a Norethynodrel–Mestranol Combination (Enovid) on Development and Growth of Carcinogen-induced Mammary Tumors in Female Rats. *Cancer*, **23**, 601.

WELSCH, C. W. & MORFORD, L. K. (1974) Influence of Chronic Treatment with 2-Bromo-α-ergocryptine (CB-154) on the Reproductive and Lactational Performance of the C3H/HeJ Female Mouse. *Experientia*, **30**, 1353.

WELSCH, C. W., NAGASAWA, H. & MEITES, J. (1970) Increased Incidence of Spontaneous Mammary Tumors in Female Rats with Induced Hypothalamic Lesions. *Cancer Res.*, **30**, 2310.

WELSCH, C. W., SQUIERS, M. D., CASSELL, E., CHEN, C. L. & MEITES, J. (1971) Median Eminence Lesions and Serum Prolactin: Influence of Ovariectomy and Ergocornine. *Am. J. Physiol.*, **221**, 1714.

YANAI, R. & NAGASAWA, H. (1970) Suppression of Mammary Hyperplastic Nodule Formation and Pituitary Prolactin Secretion in Mice Induced by Ergocornine or 2-Bromo-α-ergocryptine. *J. natn. Cancer Inst.*, **45**, 1105.